# Practice model of unit-based clinical pharmacists’ individualized daily antimicrobial use density monitoring report on antimicrobial stewardship in intensive care unit of a tertiary hospital in Guangxi, China: an interrupted time series analysis

**DOI:** 10.1186/s13756-026-01786-9

**Published:** 2026-07-02

**Authors:** Tianmin Huang, Donglan Zhu, Jun Luo, Hongliang Zhang, Yue Qiu, Yan Wen, Guoping Liu, Hanchun Wen, Taotao Liu

**Affiliations:** 1 Department of Pharmacy, The First Affiliated Hospital of Guangxi Medical University, No. 6 Shuangyong Road, Nanning, 530021 Guangxi China; 2 Department of Intensive Care Unit, The First Affiliated Hospital of Guangxi Medical University, No. 6 Shuangyong Road, Nanning, 530021 Guangxi China

**Keywords:** Antimicrobial stewardship, Intensive care units, Clinical pharmacist, Interrupted time series analysis, Antimicrobial use density

## Abstract

**Background:**

High antimicrobial exposure and antimicrobial resistance in intensive care units (ICUs) remain major challenges to patient safety. This study evaluated whether a unit-based clinical pharmacist (UBCP) model supported by an individualized daily antimicrobial use density monitoring report (IAUD-RP) could improve antimicrobial stewardship in an ICU.

**Methods:**

This single-center, retrospective, quasi-experimental study used interrupted time series analysis in a 12-bed ICU of a tertiary teaching hospital in Guangxi, China. Adult patients admitted between April 1, 2023, and October 31, 2025, were included. The intervention, initiated in August 2024, consisted of UBCP-led daily ward-wide screening, real-time risk stratification, and targeted pharmacist interventions using the individualized monitoring report. The primary outcome was antimicrobial use measured as defined daily doses (DDDs) per 100 patient-days. Secondary outcomes included average antimicrobial cost per hospitalization, multidrug-resistant organism healthcare-associated infection incidence density, clinical outcomes, and changes in specific antimicrobial agents or classes.

**Results:**

A total of 657 patients were included (295 before, 362 after). UBCP recommendations achieved a 91.7% acceptance rate. Interrupted time series analysis showed a significant immediate reduction in antimicrobial use (level change, − 29.0 DDDs/100 patient-days; *P* = 0.038), following a significant pre-intervention upward trend (+ 2.5 per month; *P* = 0.005). Interrupted time series analysis showed a significant immediate reduction in average antimicrobial cost per hospitalization (level change, − 8304 CNY; *P* = 0.035), consistent with the crude reduction (25,568 to 14,926 CNY; *P* < 0.001). Total antimicrobial consumption decreased by 11.9%. Tigecycline, quinolones, and carbapenems decreased by 52.1%, 39.7%, and 15.8%, respectively, whereas WHO Access-group antibacterial agents increased by 67.8%. After excluding patients with indeterminate (‘Others’) outcomes, clinical failure was lower post-intervention (13.0% vs. 19.2%; adjusted OR 0.62, 95% CI 0.39–0.99; *P* = 0.045).

**Conclusion:**

The UBCP-led IAUD-RP model was associated with a significant and sustained reduction in antimicrobial use density, a directionally favorable change in antimicrobial cost and prescribing pattern, and a directionally favorable but non-confirmatory signal for reduced clinical failure.

**Supplementary Information:**

The online version contains supplementary material available at 10.1186/s13756-026-01786-9.

## Introduction

Antimicrobial Resistance ranks among the top ten global health threats, contributing to around 4.71 million deaths in 2021, with 1.14 million directly attributed to it [1, 2]. Intensive Care Units (ICUs) are high-risk areas for the spread of multidrug-resistant organisms (MDROs) due to patients’ critical conditions and invasive procedures [3, 4]. MDROs prevalence in ICUs is consistently high, posing significant treatment challenges and economic burdens [5, 6]. The global Antimicrobial Stewardship (AMS) aims to combat resistance and improve patient outcomes by optimizing antimicrobial use. However, it faces challenges due to delayed data, hindering timely decisions, and lacks real-time interventions [7, 8].

China has recently emphasized the reform of pharmaceutical management and the role of pharmacists as members of multidisciplinary healthcare teams. The unit-based clinical pharmacist model has been promoted to strengthen rational medication use, patient-centered pharmaceutical care, and closer integration of pharmacists into clinical departments, as described in the Chinese expert consensus on the unit-based clinical pharmacist practice model [9]. In high-risk areas such as ICUs, this model encourages pharmacists to participate in ward rounds, medication review, and comprehensive medication-use management.

This study evaluated an ICU AMS model combining a unit-based clinical pharmacist (UBCP) with an individualized daily antimicrobial use density (AUD) monitoring report (IAUD-RP). The IAUD-RP was designed to extend routine pharmacist participation by shifting antimicrobial management from case-triggered or retrospective review to proactive daily ward-wide screening. It integrates patient-level antimicrobial exposure, organ-function indicators, current regimens, treatment duration, and pharmacist remarks into a single dashboard with visual risk labels, allowing the UBCP to rapidly prioritize high-intensity or potentially inappropriate antimicrobial use. This structure supports earlier and more targeted pharmacist–physician interventions, thereby facilitating timely regimen optimization and stewardship decision-making. Using an interrupted time series (ITS) design [10], we assessed the impact of this UBCP–IAUD-RP model on ICU antimicrobial stewardship outcomes.

## Methods

### Study design

This single-center, retrospective, quasi-experimental study using ITS analysis was conducted in the 12-bed comprehensive ICU of the First Affiliated Hospital of Guangxi Medical University, the largest tertiary grade A general hospital in Guangxi Zhuang Autonomous Region, with 2850 beds (approval No. 2026-E0022). The hospital operates several specialized ICU units, including emergency, neurology, cardiothoracic surgery, pediatric, and neonatal ICUs. The comprehensive ICU admits a heterogeneous population of adult medical and surgical patients with acute critical illness. Adult patients aged ≥ 18 years admitted between April 1, 2023, and October 31, 2025, were included, whereas those with missing key electronic data, such as antimicrobial administration records or clinical outcome data, were excluded.

### Intervention phases

The study spans 31 months, with August 2024 marking the intervention point when the UBCP practice model was implemented, as per the “Pilot Work Plan for Resident Pharmacists (Trial)” issued by the First Affiliated Hospital of Guangxi Medical University on August 1, 2024. This plan established the framework and evaluation criteria for UBCP in the ICU, ensuring their integration into the treatment team. We also reviewed antimicrobial formulary changes during the study period. Two new Reserve/Special-grade agents were introduced in the post-intervention phase: eravacycline, a novel tetracycline-class agent active against multidrug-resistant Gram-negative bacteria, and ceftobiprole medocaril, a fifth-generation cephalosporin active against resistant Gram-positive pathogens. Their cumulative use was limited, at 121.4 and 33.7 defined daily doses (DDDs), respectively, together accounting for 1.6% of total post-intervention antimicrobial DDDs. Apart from the two structured educational sessions delivered by the UBCP as part of the intervention itself (described in Sect.  2.3.1), no other formal antimicrobial-stewardship intervention or independent prescribing-education program was newly implemented in ICU during the study period; senior medical staff and routine infection-control procedures remained stable.

### Interventions and data collection

#### Study setting and UBCP staffing

The same UBCP, an associate-senior clinical pharmacist, served as the principal pharmacist throughout the 31-month study, ensuring continuity of clinical judgment and intervention authorship. After the UBCP was embedded in the ICU, structured educational sessions were delivered to ICU clinical staff on two key topics: interpretation of antimicrobial use density and strategies to reduce unnecessary antimicrobial exposure; and therapeutic drug monitoring–assisted individualized antimicrobial therapy.

One rotating clinical pharmacist trainee was assigned to ICU each year between March and July under the UBCP’s supervision. Trainee assisted only with non-clinical administrative tasks, including HIS data extraction and transcribing the UBCP’s manually calculated daily DDDs into the IAUD-RP Excel template. All clinical interventions, risk-labelling decisions, and entries in the “Remarks / Pharmacist Interventions” field were authored exclusively by the UBCP, with no clinical decision-making delegated to trainee, thereby preserving consistency of clinical judgment across the study period.

As part of routine ICU pharmacy service, the UBCP joined morning rounds, participated in selected multidisciplinary case discussions, and responded to on-call consultation requests from physicians and nurses via WeChat (Tencent, Shenzhen, China) or telephone, including both working days and non-working days. Data-entry assistance from rotating trainee, when available, helped offset the time required for IAUD-RP preparation, which was estimated at approximately 30–60 min per day.

#### IAUD-RP workflow

The IAUD-RP was designed as a one-page-per-day, ward-wide bedside dashboard that converted heterogeneous Hospital Information System (HIS) data into a standardized point-of-care stewardship tool. It was implemented as a structured Microsoft Excel^®^ workbook (Microsoft, Redmond, WA, USA) using the existing hospital information system, without additional IT infrastructure. A structured summary of the complete intervention package is provided in Supplementary Table S1.

By 17:00 every day, the ICU UBCP, with administrative assistance from the rotating trainee when present as described in Sect.  2.3.1, extracted patient-level data from the HIS for every occupied bed and entered them into the IAUD-RP template, which contained eight pre-defined columns: (i) patient identifier; (ii) age and body weight; (iii) principal diagnoses; (iv) other diagnoses; (v) liver and renal function indicators, including total bilirubin (TBIL), alanine aminotransferase (ALT), aspartate aminotransferase (AST), serum creatinine (SCr), and creatinine clearance (CrCl) estimated using the Cockcroft–Gault equation; (vi) current antimicrobial regimens, including drug name, dose, frequency, route, and start date; (vii) free-text “Remarks/Pharmacist Interventions” cell; (viii) the patient’s daily total DDDs.

For each antimicrobial agent_*i*_, the daily DDDs of patient_*j*_ were calculated as Daily DDDs_ij_ = (Daily prescribed dose_ij_) ÷ (WHO ATC/DDD reference value_i_), using the WHO ATC/DDD Index 2025 as the single reference source [15]. The UBCP manually summed these values across all concurrent antimicrobials and entered each patient’s daily total DDDs into the template. The unit-level AUD on day *t* was then computed by an embedded Excel formula at the bottom of the worksheet as AUD_t_ = (Σ_j_ Daily DDDs_j, t_) ÷ (occupied beds on day *t*) × 100. A worked example is provided in Supplementary File 1.

A three-tier visual labelling system was used to identify cases requiring stewardship attention. The UBCP manually applied red font to flag predefined liver or renal function abnormalities based on institutional laboratory reference thresholds, including elevated TBIL > 26 µmol/L, ALT > 50 U/L, AST > 40 U/L, or SCr > 106 µmol/L, and estimated CrCl < 50 mL/min or > 130 mL/min. The UBCP applied yellow cell highlighting to flag high antimicrobial exposure or complex combination therapy when an individual patient’s total daily DDDs across all concurrent antimicrobials were ≥ 2.5, or when the patient received three or more antimicrobial agents concurrently on the same day. These thresholds were selected to reduce information overload in a high-AUD ICU setting while identifying patients who might warrant prioritized stewardship review, and were applied consistently by the UBCP throughout the post-intervention period, rather than a formal written protocol. Bold formatting in the “Remarks / Pharmacist Interventions” field indicated that an actionable pharmacist recommendation had been entered. All remarks were authored exclusively by the principal UBCP and were based on drug labels, UpToDate^®^, Sanford Guide^®^, institutional treatment guidelines, drug–drug interaction screening, and local formulary availability and cost considerations.

**Fig. 1:**
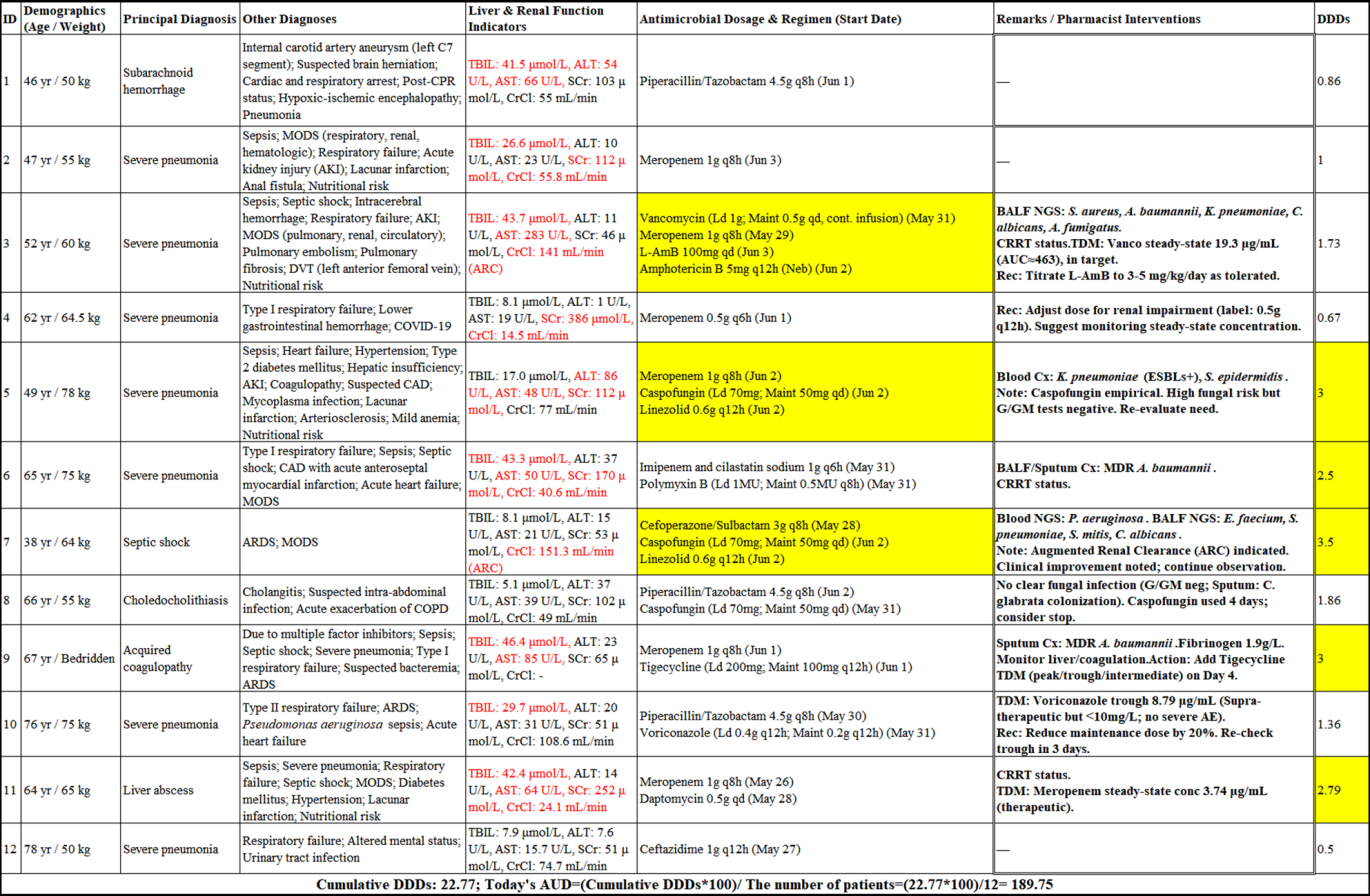
An anonymized example of the individualized daily antimicrobial use density monitoring report (IAUD-RP) The report integrates patient demographics, diagnoses, liver and renal function indicators, and antimicrobial regimens to facilitate real-time risk stratification by the Unit-based Clinical Pharmacist (UBCP). Red font indicates laboratory abnormalities, including elevated total bilirubin (TBIL), alanine aminotransferase (ALT), aspartate aminotransferase (AST), or serum creatinine (SCr), and reduced creatinine clearance (CrCl), which may require dosage adjustment. Yellow highlighting indicates high antimicrobial exposure or complex combination therapy, defined as the sum of an individual patient’s daily DDDs across all concurrent antimicrobials ≥ 2.5, or the use of three or more concurrent antimicrobial agents on the same day. “Remarks / Pharmacist Interventions” of this column functions as a comprehensive clinical log for stewardship documentation and feedback. The bottom row displays the real-time calculation of the point-prevalence AUD for the entire unit on that specific day. Abbreviations: CPR, cardiopulmonary resuscitation; MODS, multiple organ dysfunction syndrome; ARDS, acute respiratory distress syndrome; COPD, chronic obstructive pulmonary disease; CAD, coronary artery disease. Cx: Culture; NGS: Next-generation sequencing; BALF: Bronchoalveolar lavage fluid; TBIL: Total bilirubin; ALT: Alanine aminotransferase; AST: Aspartate aminotransferase; SCr: Serum creatinine; CrCl: Creatinine clearance; CRRT: Continuous renal replacement therapy; TDM: Therapeutic drug monitoring; Ld: Loading dose; Maint: Maintenance dose; Neb: Nebulization; L-AmB: Liposomal Amphotericin B; MDR: Multi-drug resistant; ESBLs: Extended-spectrum beta-lactamases; G test: 1,3-beta-D-glucan test; GM test: Galactomannan test; PCT: Procalcitonin; AUC: Area under the curve; DDDs: Defined Daily Doses; AUD: Antimicrobial use density.

The completed IAUD-RP was distributed to the ICU medical team daily via the unit’s dedicated WeChat work group and was additionally printed for the next morning’s bedside rounds. During morning rounds the UBCP discussed every flagged case directly with the attending physician and bedside nurse, recorded the consensus decision in the HIS, and updated the IAUD-RP at the next 17:00 cycle, thereby closing the daily feedback loop. An anonymized example of a completed IAUD-RP is provided as Fig. 1.

#### Recording and classification of UBCP interventions

During the post-intervention period, UBCP interventions were recorded and classified by scenario as ward-round interventions, representing active screening, or on-call consultations, representing passive responses via WeChat or telephone. Interventions were analyzed at the level of distinct pharmacist recommendations rather than unique patients; therefore, multiple recommendations for the same patient could be counted separately. Intervention types included dose optimization, adverse drug reaction, therapeutic drug monitoring, de-escalation/streamlining, antimicrobial agent selection, contraindication, interactions, discontinuation/duration. Physician acceptance was defined as implementation of the UBCP recommendation, and the acceptance rate was calculated as the number of accepted recommendations divided by the total number of recommendations.

### Outcome indicators and covariates

#### Patient baseline characteristics and covariates

Patient-level baseline characteristics were extracted from the hospital information system (HIS) for every eligible discharge and used to verify comparability between the pre- and post-intervention phases. The collected variables included: demographic characteristics (age, sex), principal diagnosis, use of organ support during the ICU stay, and length of hospital stay. The Case-Mix Index (CMI), a unit-level composite indicator derived from Diagnosis-Related Group weights assigned to discharged patients [11], was additionally computed for each phase as a measure of overall case-load complexity; higher CMI values indicate a more complex case mix.

#### Clinical outcome indicators

Clinical outcomes were assigned once per patient at the point of ICU discharge or transfer, as part of routine clinical care at that time, and were retrieved from the medical records for this retrospective analysis. At discharge, each outcome was determined independently by the attending physician and the ward Chief Resident, who classified the case separately and then reconciled their assessments to reach a final consensus, with any discrepancies resolved by discussion. Outcomes were classified using the five-category disease-outcome framework referenced in the Chinese Standard for healthcare associated infection surveillance [12], with study-specific operational definitions.

Cure was defined as complete resolution of disease-related symptoms with full functional recovery, or only minor sequelae not affecting daily life, without the need for further treatment for the index condition. Improvement indicated partial symptom relief and functional recovery, while further observation or step-down treatment was still required. Clinical failure referred to no meaningful clinical improvement, persistent symptoms, or inadequate treatment response requiring further treatment or observation. Death was defined as in-hospital death after unsuccessful resuscitation, regardless of the immediate cause. Others included outcomes not classifiable into the above categories, such as discharge for non-medical reasons or special clinical scenarios.

As a complementary microbiological indicator, the incidence density of healthcare- associated infections (HAIs) caused by specific multidrug-resistant organisms (MDROs) was calculated for each phase, following the Hospital Infection Management Medical Quality Control Indicators (2024 Edition) issued by the National Health Commission of China [13]. This patient-day–based metric is consistent with CDC/NHSN infection-surveillance reporting, in which MDRO infection incidence is calculated as HAIs by MDRO type per 1000 patient-days [14].

$$ \begin{aligned} {\mathrm{MDRO}}\;{\mathrm{HAI}}\;{\mathrm{incidence}}\;{\mathrm{density}} & = {\mathrm{number}}\;{\mathrm{of}}\;{\mathrm{new}}\;{\mathrm{HAI}}\;{\mathrm{episodes}}\;{\mathrm{caused}}\;{\mathrm{by}}\;{\mathrm{specific}}\;{\mathrm{MDROs}} \\ & \div {\mathrm{cumulative}}\;{\mathrm{inpatient}}\;{\mathrm{days}} \times {\text{1000 }} \\ \end{aligned} $$

The monitored MDROs were predefined infection-control surveillance phenotypes, including methicillin-resistant *Staphylococcus aureus* (MRSA), vancomycin-resistant *Enterococcus faecalis* or *E. faecium* (VRE), carbapenem-resistant *Klebsiella pneumoniae* (CRKP), carbapenem-resistant *Escherichia coli* (CREC), carbapenem-resistant *Pseudomonas aeruginosa* (CRPA), and carbapenem-resistant *Acinetobacter baumannii* (CRAB). Resistance phenotypes were determined according to routine antimicrobial susceptibility testing. Culture-positive MDRO findings were classified as HAIs, rather than colonization, according to institutional infection-control surveillance criteria and adjudication by the Hospital Infection Control Department. Repeated isolation of the same organism from the same infection site during the same hospitalization was counted as one episode. Organism-specific distributions are provided descriptively in Supplementary Table S2.

#### Antimicrobial utilization and cost outcomes

The primary utilization outcome was AUD, measured as DDDs per 100 patient-days, calculated as:   

$$\begin{aligned} {\text{AUD }} & {\text{ = }} \\ & \frac{{\sum {{\mathrm{DDDs}}} }} {\begin{gathered} {\text{Number of patients discharged during the same period}} \\ {\text{*average length of stay for patients discharged during the same period}}\hfill \\ \end{gathered} } \\ \end{aligned} \times 100\hfill$$

The DDD value is based on the WHO ATC/DDD Index 2025 Edition [15]. This internationally recognized standard assesses antimicrobial exposure and is essential for hospital management evaluation in China [16]. The Average Antimicrobial Cost per Hospitalization (AACPH) was the secondary utilization outcome, calculated as the total cost of antimicrobial drugs used during hospital stays divided by the number of discharged patients, in Chinese yuan (CNY). Antimicrobial cost per patient-day was calculated for each month as the monthly antimicrobial expenditure divided by the monthly occupied bed-days. All costs were expressed in nominal CNY for the study period; no inflation adjustment or price standardization was applied.

Antimicrobial use was concurrently evaluated under two parallel classification frameworks. The WHO AWaRe classification [17] groups antibacterial agents by their ecological impact on resistance selection: Access agents are recommended first-line options for common infections (target ≥ 60% of total consumption); Watch agents have a higher resistance-selection potential and are priority targets for stewardship; and Reserve agents are last-resort options preserved for confirmed extensively drug-resistant infections. The Hospital Antimicrobial Clinical Application Classification Catalogue (2023 Edition) [18] applied at our institution, by contrast, is an administrative-control framework that stratifies antimicrobials by prescribing authority: Non-restricted agents may be prescribed by any licensed physician; Restricted agents require an attending physician or above; and Special agents require an associate chief physician or above together with mandatory infectious-disease specialist consultation. Importantly, the two frameworks do not align directly. Most notably, carbapenems and glycopeptides are categorized as Watch under the WHO AWaRe schema but are elevated to Special grade under local policy. The full mapping of every antimicrobial evaluated in this study under both frameworks is provided in Supplementary Table S3.

### Statistical analysis

Data were analyzed using SPSS 26.0. Continuous variables were assessed for normality using the Shapiro–Wilk test and are presented as mean ± standard deviation or median (interquartile range), as appropriate. Between-group comparisons of baseline characteristics and outcomes were performed using the independent-samples t test or Mann–Whitney U test for continuous variables and the chi-square or Fisher’s exact test for categorical variables. MDRO HAI incidence density was expressed per 1,000 patient-days and compared using a Poisson rate test, with incidence rate ratio (IRR) reported. Antimicrobial consumption structure was quantified as monthly average defined DDDs, and pre- versus post-intervention proportions were compared using two-proportion Z-tests. UBCP interventions and acceptance outcomes were summarized descriptively and visualized using a Sankey diagram generated in Origin 2025. Clinical failure was further assessed using multivariable logistic regression adjusted for prespecified baseline covariates, with results reported as adjusted odds ratios and 95% confidence intervals. A two-sided *P* < 0.05 was considered statistically significant.

ITS analysis was performed using STATA 17. Monthly outcomes, including AUD and AACPH, were analyzed using segmented regression. Owing to the limited number of MDRO HAI events, segmented regression was not applied to this indicator. The model estimated the baseline trend, immediate level change after intervention, and post-intervention trend change, as follows: ${\mathrm{Y}}_{{\mathrm{t}}} {\text{ = }}\beta _{{\mathrm{0}}} {\text{ + }}\beta _{{\mathrm{1}}} \cdot {\mathrm{time}}_{{\mathrm{t}}} {\text{ + }}\beta _{{\mathrm{2}}} \cdot {\text{intervention + }}\beta _{{\mathrm{3}}} \cdot {\mathrm{time}}_{{{\mathrm{post}}}} {\text{ + }}\varepsilon _{{\mathrm{t}}}$. Here, $\:{\mathrm{Y}}_{\mathrm{t}}$ represents the outcome indicator for month t, $\:\mathrm{tim}{\mathrm{e}}_{\mathrm{t}}$ is the month since the study began, $\:\mathrm{intervention}$ indicates intervention status (0 before, 1 after), and $\:\mathrm{tim}{\mathrm{e}}_{\mathrm{post}}$ is the time after intervention. $\:{{\beta}}_{\mathrm{0}}$ is the initial baseline level, $\:{{\beta}}_{\mathrm{1}}$ is the pre-intervention trend, $\:{{\beta}}_{\mathrm{2}}$ is the level change at intervention, and $\:{{\beta}}_{\mathrm{3}}$ is the post-intervention trend change; $\:{{\epsilon}}_{\mathrm{t}}$ is the error term. To account for residual autocorrelation, segmented regression models were estimated using Prais–Winsten AR(1) generalized least squares with robust standard errors. Model assumptions were assessed using the Durbin–Watson statistic (autocorrelation), the Shapiro–Wilk test (residual normality), and one-way ANOVA of residuals across calendar months (seasonality). The intervention was modelled as an immediate step change, with no transition or washout period.

## Results

### Patient characteristics and case-mix indicators

A total of 657 discharged ICU patients were included, with 295 in the pre-UBCP period and 362 in the post-UBCP period. Patient characteristics were generally comparable between the two groups (Table 1). No significant differences were observed in sex, age, principal diagnoses, mechanical ventilation, continuous renal replacement therapy, extracorporeal membrane oxygenation, plasma exchange, or CMI.

**Table 1:** Patient characteristics during the pre- and post-unit-based clinical pharmacist (UBCP) intervention periods

Characteristics	Pre-UBCP	Post-UBCP	*P*-value
Discharged patient, n	295	362	
Beds, n	12	12	
Sex female, n (%)	76 (25.8%)	109 (30.1%)	0.218
Age, years	58.0 ± 16.8	59.1 ± 16.6	0.396
Principal diagnosis (top 3)			
Severe pneumonia, n (%)	74 (25.1%)	88 (24.3%)	0.890
Sepsis, n (%)	21 (7.1%)	43 (11.8%)	0.056
Sepsis shock, n (%)	17 (5.7%)	19 (5.2%)	0.908
Treatments, n (%)			
Mechanical ventilation, n (%)	270 (91.5%)	323 (89.2%)	0.392
Continuous renal replacement therapy, n (%)	101 (34.2%)	114 (31.5%)	0.508
Extracorporeal membrane oxygenation, n (%)	21 (7.1%)	34 (9.4%)	0.365
Plasma exchange, n (%)	6 (2.0%)	14 (3.9%)	0.253
Case Mix Index	3.6 ± 0.4	3.8 ± 0.4	0.098

### Categories, sources, and acceptance of UBCP recommendations

During the post-intervention period, 675 recommendation-level UBCP interventions were recorded, corresponding to 1.86 recommendations per discharged patient. Most were made during ward rounds (581/675, 86.1%), with the remainder through on-call consultations (94/675, 13.9%). Dose optimization and antimicrobial agent selection were the two most frequent categories (24.6% and 16.7%, respectively); the full breakdown by category and source is shown in Table 2. Overall, 619 of the 675 recommendations were accepted (91.7%). The acceptance rate differed by source: 525 of 581 ward-round recommendations were accepted (90.4%), whereas all on-call recommendations were accepted (94/94, 100%). The intervention pathways and acceptance flow are shown in the Sankey diagram (Figure S1)

**Table 2:** Categories, sources, and acceptance of UBCP recommendations during the post-intervention period

Category	Total, *n* (%)	Ward rounds, accepted/total (%)	On-call, accepted/total (%)	Overall acceptance, *n* (%)
Dose optimization	166 (24.6)	126/136 (92.6)	30/30 (100.0)	156 (94.0)
Antimicrobial agent selection	113 (16.7)	76/82 (92.7)	31/31 (100.0)	107 (94.7)
Adverse drug reaction monitoring	98 (14.5)	86/90 (95.6)	8/8 (100.0)	94 (95.9)
De-escalation / streamlining	96 (14.2)	73/89 (82.0)	7/7 (100.0)	80 (83.3)
Therapeutic drug monitoring	87 (12.9)	74/79 (93.7)	8/8 (100.0)	82 (94.3)
Discontinuation / duration	61 (9.0)	44/56 (78.6)	5/5 (100.0)	49 (80.3)
Drug–drug interactions	40 (5.9)	33/36 (91.7)	4/4 (100.0)	37 (92.5)
Contraindication	14 (2.1)	13/13 (100.0)	1/1 (100.0)	14 (100.0)
Total	675 (100.0)	525/581 (90.4)	94/94 (100.0)	619 (91.7)

Values are counted at the recommendation level rather than the patient level; a single patient could contribute more than one recommendation if multiple pharmacist interventions were made. UBCP: unit-based clinical pharmacist

### Overall antimicrobial stewardship and patient outcomes

Pre- and post-intervention outcomes are summarized in Table 3. AUD showed a non-significant decrease after intervention (205.1 ± 37.0 vs. 195.2 ± 29.9, *P* = 0.419), whereas AACPH decreased significantly from 25,568 ± 8629 to 14,926 ± 6560 CNY (*P* < 0.001). When expressed per patient-day, antimicrobial cost decreased from 1360.2 ± 568.5 to 1010.3 ± 368.4 CNY, although this reduction did not reach statistical significance (*P* = 0.053). MDRO HAI incidence density decreased from 4.14 to 2.34 per 1000 patient-days, corresponding to an IRR of 0.56, but this reduction was not statistically significant (*P* = 0.130). Average length of hospital stay also decreased numerically but not significantly (14.7 ± 3.4 vs. 12.8 ± 3.1 days, *P* = 0.103).

Clinical failure was lower in the post-UBCP period than in the pre-UBCP period (10.5% vs. 17.3%, *P* = 0.016), whereas cure, improvement, and death did not differ significantly. The “Others” category increased after intervention (19.3% vs. 9.8%, *P* = 0.001), predominantly owing to discharge against medical advice (Supplementary Table S4). As these outcomes are indeterminate with respect to antimicrobial response, the primary analysis excluded these patients; failure remained lower post-intervention (13.0% vs. 19.2%, *P* = 0.047). After adjustment for sex, age, mechanical ventilation, continuous renal replacement therapy, extracorporeal membrane oxygenation, and plasma exchange, the UBCP intervention remained associated with lower odds of clinical failure (adjusted OR 0.62, 95% CI 0.39–0.99; *P* = 0.045; Supplementary Table S5)

**Table 3:** Pre- versus post-intervention comparison of overall antimicrobial stewardship and patient outcomes

Outcome / indicator	Pre-UBCP	Post-UBCP	Absolute difference*	*P*-value
AUD	205.1 ± 37.0	195.2 ± 29.9	−9.9	0.419
AACPH, CNY	25,568 ± 8,629	14,926 ± 6,560	−10,642	< 0.001
Antimicrobial cost per patient-day, CNY	1360.2 ± 568.5	1010.3 ± 368.4	−349.9	0.053
MDRO HAI incidence density, per 1,000 patient-days	4.14 (23/5551)	2.34 (12/5135)	−1.80	0.130
Average length of hospital stay, days	14.7 ± 3.4	12.8 ± 3.1	−1.9	0.103
Clinical outcomes				
Cure, n (%)	2 (0.7)	0 (0)	−0.7 pp	0.201
Improvement, n (%)	142 (48.1)	179 (49.4)	+ 1.3 pp	0.798
Clinical failure, n (%)	51 (17.3)	38 (10.5)	−6.8 pp	0.016
Death, n (%)	71 (24.1)	75 (20.7)	−3.4 pp	0.304
Others, n (%)	29 (9.8)	70 (19.3)	+ 9.5 pp	0.001

*Absolute differences were calculated as post-intervention values minus pre-intervention values; AUD: antimicrobial use density; AACPH: average antimicrobial cost per hospitalization; CNY: Chinese yuan; MDRO: multidrug-resistant organisms; Antimicrobial cost per patient-day: each month as monthly antimicrobial expenditure divided by monthly occupied bed-days; HAI: healthcare-associated infection; pp: percentage points; For MDRO HAI incidence density, the incidence rate ratio was 0.56

### Changes in antimicrobial consumption structure

Changes in antimicrobial consumption structure are shown in Table 4. Total antimicrobial consumption decreased by 11.9% after UBCP implementation (744.0 to 655.7 monthly DDDs; *P* = 0.002). According to the WHO AWaRe classification, Access-category antibacterial consumption increased by 67.8% (*P* < 0.001), while Watch and Reserve consumption decreased by 13.6% and 13.5%, respectively, although these reductions were not statistically significant.

By pharmacological class, consumption decreased significantly for tigecycline (− 52.1%, *P* < 0.001), quinolones (− 39.7%, *P* < 0.001), and carbapenems (− 15.8%, *P* = 0.035). In contrast, BL/BLI combinations (+ 3.2%, *P* < 0.001), polymyxins (+ 29.0%, *P* < 0.001), and glycopeptides (+ 7.4%, *P* = 0.002) increased significantly, indicating heterogeneous redistribution across antimicrobial classes rather than uniform reductions in all high-priority agents. According to the local antimicrobial classification policy, non-restricted antimicrobial consumption increased by 54.2% (*P* < 0.001), whereas restricted and special-grade antimicrobial consumption showed non-significant decreases

**Table 4:** Pre- versus post-intervention changes in antimicrobial consumption structure (normalized by monthly average)

Dimension	Category	Pre-UBCP (monthly DDDs)	Post-UBCP (monthly DDDs)	Change (%)	Z-value	*P*-value
Overall	Total antimicrobials	744.0	655.7	− 11.9	− 3.16	0.002
Global standard (WHO AWaRe)*	Access	9.2	15.4	67.8	6.32	< 0.001
	Watch	394.2	340.5	− 13.6	− 1.37	0.171
	Reserve	189.3	163.8	− 13.5	-0.59	0.553
Pharmacological class	Tigecycline	58.0	27.8	− 52.1	− 10.8	< 0.001
(ATC classification system)	Carbapenems	222.2	187.1	− 15.8	− 2.11	0.035
	BL/BLI Combination	113.4	117.0	3.2	5.14	< 0.001
	Polymyxins	24.3	31.3	29.0	5.66	< 0.001
	Glycopeptides	28.9	31.1	7.4	3.05	0.002
	Oxazolidinones	41.5	40.3	− 3.1	1.72	0.086
	Quinolones	29.8	18	− 39.7	− 5.06	< 0.001
	Antifungals	151.3	136.1	− 10.0	0.75	0.454
	Others	74.6	67.1	− 10.1	− 1.92	0.055
Local policy	Non-restricted	7.5	11.6	54.2	4.7	< 0.001
(China Guangxi Province Grade)	Restricted	146.0	126.0	− 13.7	− 0.8	0.45
	Special	590.5	518.2	− 12.2	− 0.6	0.551

*WHO AWaRe analysis was restricted to antibacterial agents; systemic antifungal agents were excluded from the AWaRe-specific denominator and reported separately under pharmacological class; UBCP: unit-based clinical pharmacists; DDDs: defined daily doses; ATC: anatomical therapeutic chemical; BL/BLI: beta-lactam and beta-lactamase inhibitors

### Interrupted time series analysis

**Fig. 2:**
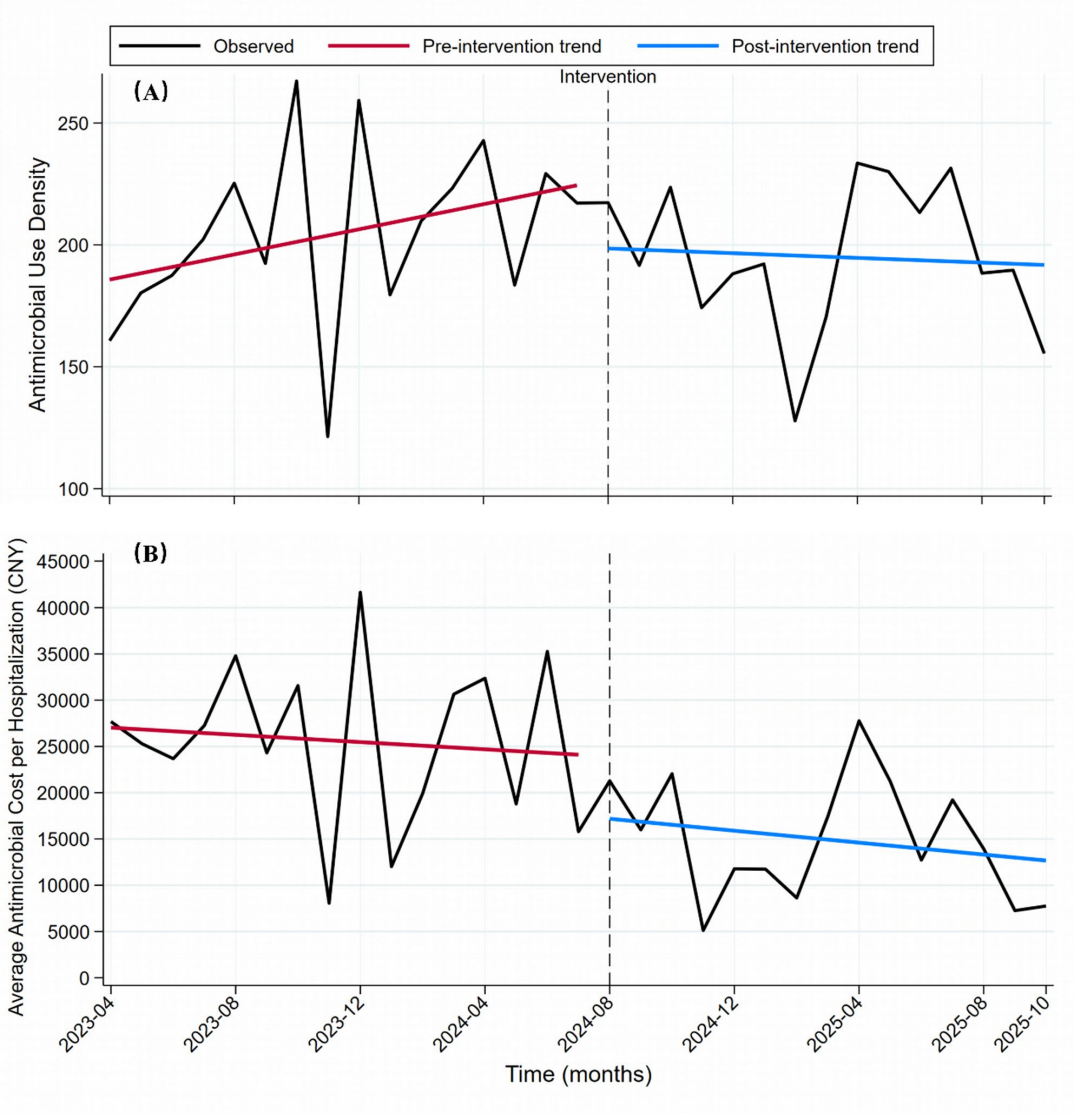
Impact of unit-based clinical pharmacist intervention on antimicrobial stewardship indicators. Interrupted time series analysis of (**A**) antimicrobial use density, (**B**) average antimicrobial cost per hospitalization; CNY: Chinese yuan. The vertical dashed line indicates the intervention point in August 2024. The solid black line represents the observed monthly data. The solid red and blue lines represent the fitted trends before and after the intervention, respectively. The discontinuity between the fitted pre- and post-intervention lines represents the estimated immediate level change from the segmented regression model; no transition or washout period was applied.

ITS analysis was performed for AUD and AACPH (Table 5; Fig. 2). Durbin–Watson statistics were 1.82 for AUD and 1.93 for AACPH, indicating no substantial first-order residual autocorrelation. Residuals were consistent with normality (Shapiro–Wilk *P* = 0.605 for AUD and 0.995 for AACPH), and one-way ANOVA of residuals across calendar months showed no significant seasonality (*P* = 0.40 and 0.068, respectively); full residual diagnostics are provided in Supplementary Figures S2 (AUD) and S3 (AACPH)

**Table 5:** Parameter estimates of the interrupted time series analysis for antimicrobial stewardship indicators

Indicators	Intercept		Baseline trend		Level change		Trend change		Model fit	
	β0 (95% CI)	*P*-value	β1 (95% CI)	*P*-value	β2(95% CI)	*P*-value	β3(95% CI)	*P*-value	DW	*R* ^2^
AUD	184.2 (163.1 to 205.3)	< 0.001	2.5 (0.8 to 4.3)	0.005	− 29.0 (− 56.2 to − 1.7)	0.038	− 2.6 (− 5.7 to 0.5)	0.093	1.82	0.36
AACPH	26,761 (22522 to30999)	< 0.001	− 122.0 (-592.7 to 348.6)	0.599	−8304 (− 15984.8 to − 623.7)	0.035	−85.5 (− 805.7 to 634.6)	0.809	1.93	0.52

AUD: antimicrobial use density; AACPH: average antimicrobial cost per hospitalization; CI: confidence interval; DW: durbin–Watson statistic for first-order residual autocorrelation; R²: coefficient of determination for model fit

For AUD, the pre-intervention trend showed a significant monthly increase (β₁ = 2.5, *P* = 0.005). The intervention was associated with a significant immediate level reduction in AUD (β₂ = −29.0, 95% CI − 56.2 to − 1.7; *P* = 0.038), while the post-intervention trend change was negative but not statistically significant (β₃ = −2.6, *P* = 0.093)

For AACPH, the pre-intervention trend was not significant (β1 = − 122.0, *P* = 0.599). The intervention was associated with a significant immediate level reduction (β2 = − 8304 CNY, 95% CI − 15984.8 to − 623.7; *P* = 0.035), with no significant post-intervention trend change (β3 = − 85.5, *P* = 0.809).

## Discussion

The IAUD-RP is a nudge intervention aimed at overcoming clinical inertia. Unlike rigid electronic medical record alerts, it uses visual risk stratification to modify decision-making at the point of care [19]. The study’s IAUD-RP was manually compiled by UBCP, not through an automated IT system. Despite the growing prevalence of AI and intelligent technology, automated systems often experience alarm fatigue due to their inability to assess clinical context, and potentially result in the oversight of critical notifications [20, 21]. IAUD-RP offers distinct benefits by requiring UBCP to actively engage with patient infection data, enhancing their ability to detect subtle drug treatment issues beyond automated reports. While the tool itself (a structured Excel workbook on existing HIS data) does not require additional IT infrastructure, sustained implementation does require dedicated pharmacist time and specialized clinical expertise.

Additionally, this practice model enhances intervention accuracy and doctor compliance [22–24]. A study at a French university hospital revealed that pharmacy residents mainly worked on optimizing prescriptions, achieving an 89.3% acceptance rate, with 44.4% of interventions initiated by the ICU team [25]. Albayrak A., et al. reported that dose changes accounted for 56.79% of planned interventions, with a 90.8% acceptance and full implementation rate [26]. However, in our research, the high overall acceptance rate of pharmacist recommendations (91.7%), including 100% acceptance for on-call consultations, should be interpreted cautiously. Most recommendations involved technically focused issues, such as dose adjustment, therapeutic drug monitoring, and drug–drug interaction screening, where pharmacists have clear professional expertise and physician acceptance is relatively straightforward. Acceptance may be lower for more controversial recommendations, such as discontinuing empirical broad-spectrum therapy or declining escalation requests. In addition, the collaborative hierarchy of Chinese tertiary hospitals, in which UBCPs participate in ward rounds under the same departmental leadership as prescribing physicians, may have facilitated acceptance and may not be fully generalizable to other settings. Finally, acceptance was not adjudicated blindly, which may have introduced measurement bias. Therefore, the 91.7% acceptance rate should be viewed as high uptake of pharmacist recommendations in this specific institutional context, rather than direct evidence of equivalent clinical effectiveness.

IAUD-RP intervention offers a distinct advantage over traditional stewardship models by providing immediate feedback [5]. By daily visualizing each patient’s antimicrobial burden, it prompts clinicians to promptly eliminate unnecessary treatments, leading to the rapid decline observed in our model. Before the intervention, there was a significant increase in AUD (slope = 2.5, *P* = 0.005), indicating a trend towards excessive use without supervision. After implementing the intervention, there was a significant immediate reduction in AUD (level change = -29.0, *P* = 0.038), highlighting the role of UBCP in reducing unnecessary prescriptions. This result aligns with earlier research demonstrating the link between pharmacist-led multifaceted antimicrobial stewardship programmes and decreased antimicrobial usage [27, 28].

Both the crude comparison and the ITS analysis showed a significant reduction in AACPH after the intervention. One possible explanation is that China’s National Volume-Based Procurement (VBP) policy reduced certain antimicrobial prices during the study period, which may have increased the variance of monthly cost data [29, 30]. This external price effect, which can confound the estimated effect of the primary intervention [31], cannot be fully separated from the stewardship effect and should be regarded as a hypothesis rather than an established cause. Because AUD is derived from defined daily doses rather than prices, the VBP policy is not expected to have materially affected the primary AUD analysis. In addition, the reduction in cost per patient-day did not reach statistical significance (*P* = 0.053), indicating that part of the per-hospitalization reduction reflects shorter length of stay rather than lower daily antimicrobial intensity alone. The key finding is the significant decrease in AACPH without affecting patient safety. Clinical failure showed a directionally favorable but non-confirmatory signal, and all-cause mortality remained unchanged, countering concerns that strict stewardship might lead to under-treatment of critically ill patients [32]. The non-significant ITS finding for MDRO HAI incidence density was expected, as resistance-related outcomes usually change over longer time horizons [33]. A 15-month observation period may not adequately capture changes in drug-resistant bacterial populations. Additionally, MDROs spread is heavily influenced by infection control practices like hand hygiene and isolation, making it unlikely that a single drug management strategy will quickly alter the situation [34].

The intervention was associated with a potentially favorable shift in antimicrobial use. Access-category agents increased by 67.8% according to the WHO AWaRe classification, indicating greater use of preferred first- or second-line agents. Although the reductions in Watch and Reserve agents were not statistically significant, these findings are consistent with a directional improvement in antimicrobial selection. The use of tigecycline and carbapenems decreased by 52.1% and 15.8%, respectively. Under the local antimicrobial classification policy, the UBCP incorporated the approval and review of “special-use” antimicrobials into daily IAUD-RP–based stewardship activities, translating administrative restrictions into practical bedside drug-selection criteria. However, the increases in polymyxin and glycopeptide use, by 29.0% and 7.4%. They may reflect pathogen-directed therapy for carbapenem-resistant Gram-negative infections or MRSA after susceptibility results became available [35–38]. Alternatively, they may represent compensatory escalation after reductions in carbapenem or tigecycline use, or changes in resistance ecology. Although Supplementary Table S2 showed no apparent worsening in major MDRO patterns [39], these findings were descriptive and limited by small event numbers. Thus, increased polymyxin and glycopeptide use should be viewed as a signal requiring continued monitoring rather than as unequivocal evidence of improved stewardship. Eravacycline and ceftobiprole medocaril were introduced post-intervention but contributed only 1.6% of total antimicrobial DDDs, making a material impact on AUD, cost, or MDRO trends unlikely.

The post-intervention increase in the ‘Others’ category (9.8% to 19.3%, *P* = 0.001) was driven predominantly by discharge against medical advice (24 to 59 cases), a common phenomenon in Chinese ICUs reflecting family-initiated treatment withdrawal for financial, social, and cultural reasons [40]. Because the antimicrobial response in these patients cannot be ascertained at discharge, we repeated the analysis excluding them. Given this indeterminacy and the absence of severity adjustment, the clinical-failure finding should be regarded as supportive rather than confirmatory.

This study has several limitations. As a single-center, retrospective, interrupted time-series study without a concurrent control group, it can characterize the temporal association between the intervention and changes in antimicrobial use but cannot establish causation; co-occurring secular trends and contemporaneous policy changes may have contributed to the observed changes. Patient-level analyses were adjusted for available covariates but could not incorporate validated severity scores (e.g., SOFA, APACHE II) or physiological markers (vasopressor use, serum lactate); residual confounding by illness severity therefore remains, and—together with the fact that outcome assessors were not blinded to the intervention period—clinical-outcome findings should be interpreted cautiously. Antimicrobial use was quantified using DDDs which may underestimate exposure in critically ill patients (e.g., with renal replacement, obesity, or severe sepsis); days of therapy would complement DDD but the required records were inaccessible for the full period. The relatively short observation period and limited number of MDRO HAI events reduced the power to detect resistance-related changes. In addition, the model’s reliance on a single principal UBCP with both clinical-pharmacy training and antimicrobial-stewardship expertise is itself a limitation for broader implementation. Future multi-ICU or multicenter prospective studies should incorporate more granular severity measures, patient-indexed intervention records, and formal agreement testing.

## Conclusion

In this single-center ICU study, the IAUD-RP–supported UBCP model was associated with reduced AUD, lower antimicrobial cost per hospitalization, and a more favorable antimicrobial use pattern. Clinical failure showed a directionally favorable but non-confirmatory signal, whereas MDRO-related outcomes showed only non-significant favorable trends. These findings suggest potential benefits for antimicrobial prescribing in high-AUD ICU settings, but broader implementation should consider workflow requirements and pharmacist-dependent judgment.

## Supplementary Information

Below is the link to the electronic supplementary material.

Supplementary Material 1

Supplementary Material 2

Supplementary Material 3

Supplementary Material 4

Supplementary Material 5

Supplementary Material 6

Supplementary Material 7

Supplementary Material 8

Supplementary Material 9

## Data Availability

The datasets used and/or analysed during the current study are available from the corresponding author on reasonable request.

## Abbreviations

AACPH: Average antimicrobial cost per hospitalization
ALT: Alanine aminotransferase
AMS: Antimicrobial stewardship
ARIMA: Autoregressive integrated moving average
AST: Aspartate aminotransferase
ATC: Anatomical therapeutic chemical
AUD: Antimicrobial use density
AWaRe: Access, Watch, Reserve
BL/BLI: Beta-lactam and beta-lactamase inhibitor
CDC: Centers for disease control and prevention
CI: Confidence interval
CMI: Case-Mix Index
CNY: Chinese yuan
CrCl: Creatinine clearance
CRAB: Carbapenem-resistant *Acinetobacter baumannii*
CREC: Carbapenem-resistant *Escherichia coli*
CRKP: Carbapenem-resistant *Klebsiella pneumoniae*
CRPA: Carbapenem-resistant *Pseudomonas aeruginosa*
CRRT: Continuous renal replacement therapy
DDDs: Defined daily doses
DW: Durbin–Watson
HAI: Healthcare-associated infection
HAIs: Healthcare-associated infections
HIS: Hospital information system
IAUD-RP: Individualized daily antimicrobial use density monitoring report
ICU: Intensive care unit
ICUs: Intensive care units
IRR: Incidence rate ratio
ITS: Interrupted time series
MDRO: Multidrug-resistant organism
MDROs: Multidrug-resistant organisms
MRSA: Methicillin-resistant *Staphylococcus aureus*
NHSN: National healthcare safety network
OR: Odds ratio
R²: Coefficient of determination
SCr: Serum creatinine
TBIL: Total bilirubin
TDM: Therapeutic drug monitoring
UBCP: Unit-based clinical pharmacist
VBP: Volume-based procurement
VRE: Vancomycin-resistant *Enterococcus faecalis* or *Enterococcus faecium*
WHO: World Health Organization
