# Supplementary material for: Practice model of unit-based clinical pharmacists’ individualized daily antimicrobial use density monitoring report on antimicrobial stewardship in intensive care unit of a tertiary hospital in Guangxi, China: an interrupted time series analysis
Source: Antimicrob Resist Infect Control. 2026 Jul 2;15:96. doi: 10.1186/s13756-026-01786-9 (PMC13411574; doi:10.1186/s13756-026-01786-9)
Supplement: Supplementary file 2 — Supplementary Material 2 [file 13756_2026_1786_MOESM2_ESM.docx]

## Supplementary Table S1. UBCP–IAUD-RP intervention package: implementation checklist for replicability

| Component | Specification (as implemented) |
| --- | --- |
| Personnel — who | One principal unit-based clinical pharmacist (UBCP) with both clinical-pharmacy and antimicrobial-stewardship training, embedded in the ICU. A rotating pharmacy trainee provided administrative assistance with HIS data extraction when present. All pharmacist recommendations were authored exclusively by the principal UBCP. |
| Tool — what | A one-page-per-day, ward-wide bedside dashboard (Individualized Antimicrobial-Use Daily Report, IAUD-RP), built as a structured Microsoft Excel® workbook on the existing HIS; no additional IT infrastructure required. |
| Frequency — how often | Daily. Data extracted and entered by 17:00 each day for every occupied bed; flagged cases discussed at the next morning bedside round; report updated at the following 17:00 cycle (daily closed feedback loop). |
| Report generation — how | UBCP extracts patient-level HIS data into an 8-column template: (i) patient identifier; (ii) age/body weight; (iii) principal diagnoses; (iv) other diagnoses; (v) liver/renal indicators (TBIL, ALT, AST, SCr, Cockcroft–Gault CrCl); (vi) current antimicrobial regimen (drug, dose, frequency, route, start date); (vii) Remarks/Pharmacist Interventions; (viii) patient daily total DDDs. Daily DDDs per agent = prescribed dose ÷ WHO ATC/DDD reference value (WHO ATC/DDD Index 2025). Unit AUD = (Σ patient daily DDDs ÷ occupied beds) × 100, via embedded Excel formula. |
| Risk labelling — how cases are flagged | Three-tier visual system applied consistently by the UBCP: (1) RED font — liver/renal abnormality (TBIL >26 μmol/L, ALT >50 U/L, AST >40 U/L, SCr >106 μmol/L, or CrCl <50 or >130 mL/min); (2) YELLOW highlight — high exposure/complex therapy (an individual patient’s total daily DDDs ≥2.5, or ≥3 concurrent antimicrobials); (3) BOLD in Remarks — an actionable pharmacist recommendation has been entered. |
| Communication — how recommendations reach the team | Completed IAUD-RP distributed daily via the ICU dedicated WeChat work group and printed for the next morning round. During rounds the UBCP discusses every flagged case directly with the attending physician and bedside nurse; consensus decision recorded in the HIS. |
| Intervention recording | Recommendations classified by scenario (ward-round = active screening; on-call = passive WeChat/telephone) and by type (dose optimization, ADR, TDM, de-escalation/streamlining, agent selection, contraindication, interactions, discontinuation/duration). Acceptance = physician implementation; acceptance rate = accepted ÷ total recommendations. |
| Knowledge resources required | Drug labels, UpToDate®, Sanford Guide®, institutional treatment guidelines, drug–drug interaction screening, and local formulary availability/cost information. |
| Training / expertise required for replication | A pharmacist with combined clinical-pharmacy and antimicrobial-stewardship competency, familiarity with WHO ATC/DDD methodology and Cockcroft–Gault estimation, and basic spreadsheet skills. The model required no additional IT infrastructure beyond existing HIS access and standard office software, although it relied on the subscription-based clinical knowledge resources listed above. |

*UBCP: unit-based clinical pharmacist; IAUD-RP: individualized daily antimicrobial use density monitoring report; HIS: hospital information system; AUD: antimicrobial use density; DDD: defined daily dose; TDM: therapeutic drug monitoring; ADR: adverse drug reaction; CrCl: creatinine clearance; TBIL: Total bilirubin; ALT: Alanine aminotransferase; AST: Aspartate aminotransferase; SCr: Serum creatinine; CrCl: Creatinine clearance; ATC：Anatomical Therapeutic Chemical Classification System.*
