# Supplementary material for: Practice model of unit-based clinical pharmacists’ individualized daily antimicrobial use density monitoring report on antimicrobial stewardship in intensive care unit of a tertiary hospital in Guangxi, China: an interrupted time series analysis
Source: Antimicrob Resist Infect Control. 2026 Jul 2;15:96. doi: 10.1186/s13756-026-01786-9 (PMC13411574; doi:10.1186/s13756-026-01786-9)
Supplement: Supplementary file 3 — Supplementary Material 3 [file 13756_2026_1786_MOESM3_ESM.docx]

**Supplementary Table S4.** Composition of the "Others" clinical-outcome category during the pre- and post-unit-based clinical pharmacist intervention periods

| Disposition category | Pre-UBCP, n (%) | Post-UBCP, n (%) | Change |
| --- | --- | --- | --- |
| Discharge against medical advice (DAMA) | 24 (8.1) | 59 (16.3) | +35 |
| Administrative transfer | 5 (1.7) | 8 (2.2) | +3 |
| Transfer for end-of-life care | 0 (0.0) | 3 (0.8) | +3 |
| Total | 29 (9.8) | 70 (19.3) | +41 |

**Footnote.**

*• Percentages are of total discharges during the pre- and post-unit-based clinical pharmacist intervention periods (pre n=295; post n=362). The post-intervention increase was driven predominantly by DAMA. Within the 'Others' category, DAMA accounted for the majority in both phases (24/29, 82.8% pre; 59/70, 84.3% post).*
