# Supplementary material for: Practice model of unit-based clinical pharmacists’ individualized daily antimicrobial use density monitoring report on antimicrobial stewardship in intensive care unit of a tertiary hospital in Guangxi, China: an interrupted time series analysis
Source: Antimicrob Resist Infect Control. 2026 Jul 2;15:96. doi: 10.1186/s13756-026-01786-9 (PMC13411574; doi:10.1186/s13756-026-01786-9)
Supplement: Supplementary file 4 — Supplementary Material 4 [file 13756_2026_1786_MOESM4_ESM.docx]

**Supplementary Table S3.** Classification of antimicrobials evaluated in this study under the WHO AWaRe (2025) framework and *Classification Management Catalogue of Antimicrobial Drug Clinical Application in Guangxi Zhuang Autonomous Region (2023 Edition)* applied at the study institution

| **Antimicrobial agent** | **ATC code** | **WHO AWaRe (2025)** | **Local grade (2023)** | **DDD value** |
| --- | --- | --- | --- | --- |
| ***Penicillins*** | | | | |
| Ampicillin / sulbactam | J01CR01 | Access | Restricted | 6 g (P) |
| Piperacillin / tazobactam | J01CR05 | Watch | Restricted | 14 g (P) |
| Piperacillin / sulbactam | J01CR05 | Watch | Restricted | 14 g (P) |
| ***Cephalosporins (1st gen)*** | | | | |
| Cefazolin | J01DB04 | Access | Non-restricted | 3 g (P) |
| ***Cephalosporins (2nd gen) / Cephamycins*** | | | | |
| Cefuroxime | J01DC02 | Watch | Non-restricted | 3 g (P) |
| Cefoxitin | J01DC01 | Watch | Restricted | 6 g (P) |
| Cefmetazole | J01DC09 | Watch | Restricted | 4 g (P) |
| ***Cephalosporins (3rd gen)*** | | | | |
| Ceftriaxone | J01DD04 | Watch | Non-restricted | 2 g (P) |
| Ceftazidime | J01DD02 | Watch | Restricted | 4 g (P) |
| Cefodizime | J01DD09 | Watch | Restricted | 2 g (P) |
| ***Cephalosporins (5th gen)*** | | | | |
| Ceftobiprole medocaril | J01DI01 | Reserve | Special | 1.5 g (P) |
| ***Cephalosporin / β-lactamase inhibitor combinations*** | | | | |
| Cefoperazone / sulbactam | J01DD62 | Watch | Restricted | 4 g (P) |
| Ceftazidime / avibactam | J01DD52 | Reserve | Special | 6 g (P) |
| ***Other β-lactams (Monobactams)*** | | | | |
| Aztreonam | J01DF01 | Reserve | Restricted | 4 g (P) |
| ***Carbapenems*** | | | | |
| Imipenem / cilastatin | J01DH51 | Watch | Special | 2 g (P) |
| Meropenem | J01DH02 | Watch | Special | 3 g (P) |
| Biapenem | J01DH05 | Watch | Special | 1.2 g (P) |
| ***Tetracyclines (extended-spectrum) / Glycylcyclines / Aminomethylcyclines*** | | | | |
| Doxycycline (oral) | J01AA02 | Access | Non-restricted | 0.1 g (O) |
| Doxycycline (IV) | J01AA02 | Access | Restricted | 0.1 g (P) |
| Tigecycline | J01AA12 | Reserve | Special | 0.1 g (P) |
| Eravacycline | J01AA13 | Reserve | Special | 0.14 g (P) |
| Omadacycline (IV) | J01AA15 | Reserve | Special | 0.1 g (P) |
| ***Macrolides*** | | | | |
| Azithromycin (IV) | J01FA10 | Watch | Restricted | 0.5 g (P) |
| ***Lincosamides*** | | | | |
| Clindamycin (IV) | J01FF01 | Access | Restricted | 1.8 g (P) |
| ***Aminoglycosides*** | | | | |
| Amikacin | J01GB06 | Access | Non-restricted | 1 g (P) |
| ***Fluoroquinolones*** | | | | |
| Ciprofloxacin (IV) | J01MA02 | Watch | Restricted | 0.8 g (P) |
| Levofloxacin (IV) | J01MA12 | Watch | Non-restricted | 0.5 g (P) |
| Moxifloxacin (IV) | J01MA14 | Watch | Restricted | 0.4 g (P) |
| ***Glycopeptides*** | | | | |
| Vancomycin | J01XA01 | Watch | Special | 2 g (P) |
| Teicoplanin | J01XA02 | Watch | Special | 0.4 g (P) |
| ***Polymyxins*** | | | | |
| Polymyxin B sulfate | J01XB02 | Reserve | Special | 0.15 g (P) |
| Colistimethate sodium (CMS) | J01XB01 | Reserve | Special | 9 MU (P) |
| ***Nitroimidazoles*** | | | | |
| Metronidazole (IV) | J01XD01 | Access | Non-restricted | 1.5 g (P) |
| ***Oxazolidinones*** | | | | |
| Linezolid (oral) | J01XX08 | Reserve | Restricted | 1.2 g (O) |
| Linezolid (IV) | J01XX08 | Reserve | Special | 1.2 g (P) |
| ***Lipopeptides*** | | | | |
| Daptomycin | J01XX09 | Reserve | Special | 0.28 g (P) |
| ***Other antibacterials*** | | | | |
| Fosfomycin sodium (IV) | J01XX01 | Reserve | Restricted | 8 g (P) |
| ***Antifungals (systemic) — Triazoles*** | | | | |
| Fluconazole (oral) | J02AC01 | — | Non-restricted | 0.2 g (O) |
| Fluconazole (IV) | J02AC01 | — | Restricted | 0.2 g (P) |
| Voriconazole (oral) | J02AC03 | — | Restricted | 0.4 g (O) |
| Voriconazole (IV) | J02AC03 | — | Special | 0.4 g (P) |
| Posaconazole (IV) | J02AC04 | — | Special | 0.3 g (P) |
| Isavuconazole (as isavuconazonium sulfate, IV) | J02AC05 | — | Special | 0.2 g (P) |
| ***Antifungals (systemic) — Echinocandins*** | | | | |
| Caspofungin | J02AX04 | — | Special | 0.05 g (P) |
| ***Antifungals (systemic) — Polyenes*** | | | | |
| Amphotericin B (conventional / deoxycholate) | J02AA01 | — | Special | 0.035 g (P) |
| Amphotericin B (cholesteryl sulfate complex) | J02AA01 | — | Special | 0.21 g (P) |
| Amphotericin B (liposomal) | J02AA01 | — | Special | 0.21 g (P) |
| ***Antifungals (systemic) — Pyrimidine analogues*** | | | | |
| Flucytosine (IV) | J02AX01 | — | Non-restricted | 10 g (P) |

**Footnotes.**

*• ATC: Anatomical Therapeutic Chemical classification system (WHO Collaborating Centre for Drug Statistics Methodology, 2026 update; available at https://atcddd.fhi.no/atc_ddd_index/).*

*• DDD route designators: O = oral; P = parenteral; MU = million International Units (IU).*

*• WHO AWaRe categories per the 2025 WHO Model List of Essential Medicines: Access (first/second-line empirical agents for common syndromes); Watch (key agents at higher resistance-selection risk, prioritized for stewardship); Reserve (last-resort options preserved for confirmed multidrug-resistant infections).*

*• Local grades from Classification Management Catalogue of Antimicrobial Drug Clinical Application in Guangxi Zhuang Autonomous Region (2023 Edition) applied at the study institution: Non-restricted (any licensed physician may prescribe); Restricted (attending physician or above only); Special (associate chief physician or above + mandatory infectious-disease specialist consultation).*

*• "—" in the WHO AWaRe column denotes that the agent is not classified within the AWaRe framework: Antifungal agents (J02 group: triazoles, echinocandins, polyenes, pyrimidine analogues).*

*• Key divergence from WHO AWaRe: agents labelled "Watch" by WHO AWaRe but elevated to "Special" by local policy include all carbapenems, both glycopeptides, and selected fluoroquinolones (when administered intravenously). This reflects a deliberate local policy decision to impose tighter administrative control on a broader range of high-priority agents than the WHO ecological framework would suggest.*
