# Supplementary material for: Practice model of unit-based clinical pharmacists’ individualized daily antimicrobial use density monitoring report on antimicrobial stewardship in intensive care unit of a tertiary hospital in Guangxi, China: an interrupted time series analysis
Source: Antimicrob Resist Infect Control. 2026 Jul 2;15:96. doi: 10.1186/s13756-026-01786-9 (PMC13411574; doi:10.1186/s13756-026-01786-9)
Supplement: Supplementary file 5 — Supplementary Material 5 [file 13756_2026_1786_MOESM5_ESM.docx]

**Supplementary Table S5.** Multivariable logistic regression analysis for clinical failure (primary analysis: patients with indeterminate "Others" outcomes excluded)

| Variable | Adjusted OR | 95% CI | P-value |
| --- | --- | --- | --- |
| UBCP intervention, post- vs pre- UBCP | 0.622 | 0.392–0.989 | 0.045 |
| Female sex, vs Male | 1.414 | 0.858–2.328 | 0.174 |
| Age, per 1-year increase | 1.001 | 0.988–1.015 | 0.840 |
| Mechanical ventilation, yes vs no | 1.450 | 0.588–3.577 | 0.420 |
| Continuous renal replacement therapy, yes vs no | 1.265 | 0.779–2.055 | 0.341 |
| Extracorporeal membrane oxygenation, yes vs no | 1.849 | 0.885–3.860 | 0.102 |
| Plasma exchange, yes vs no | 1.160 | 0.312–4.318 | 0.825 |

**Footnote.**

*•Clinical failure was coded as a binary outcome (1 vs all other outcomes coded 0). The primary analysis excluded the 99 patients with indeterminate ("Others") outcomes, leaving 558 patients. Seven covariates were prespecified on the basis of clinical relevance and established prognostic importance in critically ill patients, and were entered simultaneously. Reference categories were the pre-UBCP period, male sex, and absence of the corresponding treatment. OR >1 indicates higher odds of clinical failure. All variance inflation factors <1.1; Hosmer-Lemeshow P=0.568. For comparison, the same model in the full cohort (n=657, including "Others") yielded an adjusted OR for UBCP intervention of 0.545 (95% CI 0.345–0.862, P=0.009). UBCP: unit-based clinical pharmacist; OR: odds ratio; CI: confidence interval.*
