# Supplementary material for: Practice model of unit-based clinical pharmacists’ individualized daily antimicrobial use density monitoring report on antimicrobial stewardship in intensive care unit of a tertiary hospital in Guangxi, China: an interrupted time series analysis
Source: Antimicrob Resist Infect Control. 2026 Jul 2;15:96. doi: 10.1186/s13756-026-01786-9 (PMC13411574; doi:10.1186/s13756-026-01786-9)
Supplement: Supplementary file 6 — Supplementary Material 6 [file 13756_2026_1786_MOESM6_ESM.docx]

**Supplementary Table S2.** Pathogen-specific multidrug-resistant organism (MDRO) burden in the ICU during the pre- and post-intervention phases

| Pathogen | Pre-UBCP MDR / total cultures (% MDR) | Post-UBCP MDR / total cultures (% MDR) |
| --- | --- | --- |
| *Acinetobacter baumannii* (CRAB) | 85 / 93 (91.4%) | 78 / 97 (80.4%) |
| *Klebsiella pneumoniae* (CRKP) | 17 / 43 (39.5%) | 14 / 32 (43.8%) |
| *Pseudomonas aeruginosa* (CRPA) | 17 / 38 (44.7%) | 10 / 29 (34.5%) |
| *Staphylococcus aureus* (MRSA) | 9 / 13 (69.2%) | 5 / 16 (31.2%) |
| *Escherichia coli* (CREC) | 5 / 25 (20.0%) | 6 / 20 (30.0%) |
| *Enterococcus spp.* (VRE) | 0 / 27 (0.0%) | 1 / 31 (3.2%) |
| Total (six MDRO species) | **133 / 239 (55.6%)** | **114 / 225 (50.7%)** |

**Footnote.**

*• Cultures were de-duplicated according to the standard hospital epidemiology rule: when the same MDRO species was repeatedly isolated from the same patient during a single hospitalization, only the first isolate was counted.*

*• CRAB: carbapenem-resistant A. baumannii; CRKP: carbapenem-resistant K. pneumoniae; CRPA: carbapenem-resistant P. aeruginosa; MRSA: methicillin-resistant S. aureus; CREC: carbapenem-resistant E. coli; VRE: vancomycin-resistant Enterococcus; PD: patient-days.*
