# Supplementary material for: Practice model of unit-based clinical pharmacists’ individualized daily antimicrobial use density monitoring report on antimicrobial stewardship in intensive care unit of a tertiary hospital in Guangxi, China: an interrupted time series analysis
Source: Antimicrob Resist Infect Control. 2026 Jul 2;15:96. doi: 10.1186/s13756-026-01786-9 (PMC13411574; doi:10.1186/s13756-026-01786-9)
Supplement: Supplementary file 7 — Supplementary Material 7 [file 13756_2026_1786_MOESM7_ESM.docx]

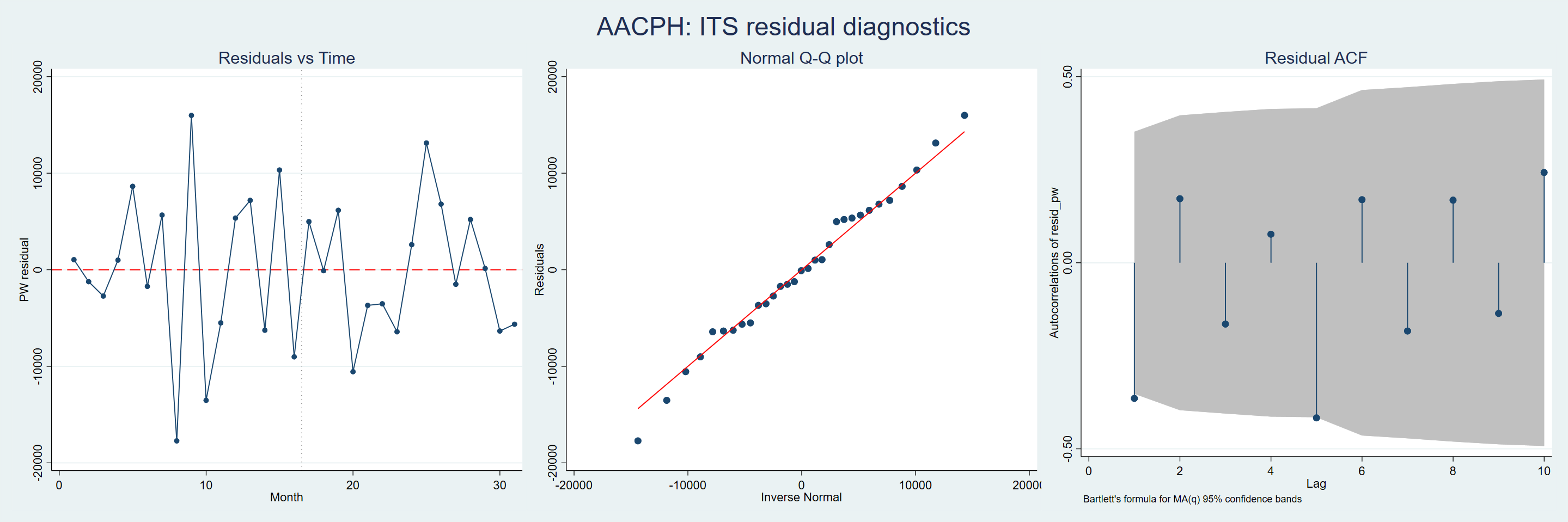


**Supplementary Figure S3.** Residual diagnostics for the Prais–Winsten AR(1) interrupted time-series model of average antimicrobial cost per hospitalization (AACPH); panels as in Supplementary Figure S2. Residuals were randomly scattered around zero (A); the Q–Q plot showed close agreement with the reference line, consistent with normal residuals (Shapiro–Wilk *P* = 0.995) (B); and all autocorrelations fell within the Bartlett 95% bands, indicating no residual autocorrelation after AR(1) correction (transformed Durbin–Watson = 1.93, ρ = −0.37) (C). One-way ANOVA of residuals by calendar month showed no significant seasonality (*F*(11,19) = 2.16, *P* = 0.068).
