# Supplementary material for: Practice model of unit-based clinical pharmacists’ individualized daily antimicrobial use density monitoring report on antimicrobial stewardship in intensive care unit of a tertiary hospital in Guangxi, China: an interrupted time series analysis
Source: Antimicrob Resist Infect Control. 2026 Jul 2;15:96. doi: 10.1186/s13756-026-01786-9 (PMC13411574; doi:10.1186/s13756-026-01786-9)
Supplement: Supplementary file 8 — Supplementary Material 8 [file 13756_2026_1786_MOESM8_ESM.docx]

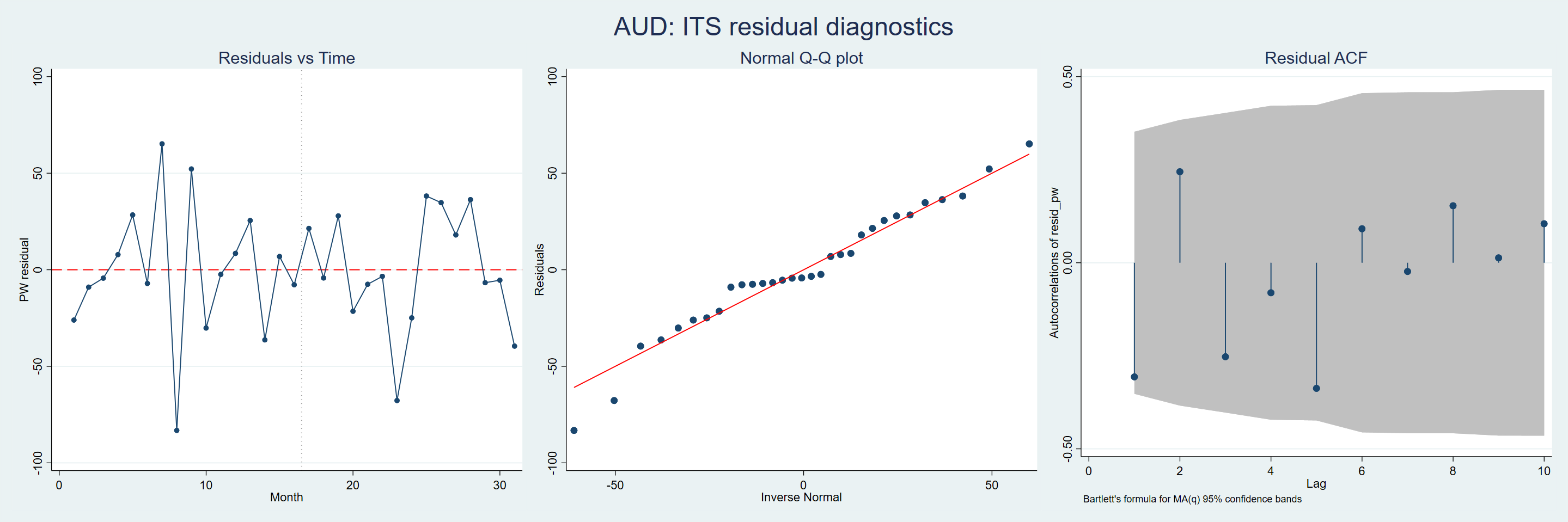


**Supplementary Figure S2.** Residual diagnostics for the Prais–Winsten AR(1) interrupted time-series model of antimicrobial use density (AUD). **(A)** Residuals versus time; the red dashed line marks zero and the grey dotted line the intervention point (month 17, August 2024); residuals were randomly scattered around zero with no systematic trend or change in variance between periods. **(B)** Normal quantile–quantile plot; points lay close to the reference line, consistent with approximately normal residuals (Shapiro–Wilk *P* = 0.605). **(C)** Autocorrelation function with Bartlett 95% confidence bands; all autocorrelations, including lag 1, fell within the bands, indicating no residual first-order autocorrelation after AR(1) correction (transformed Durbin–Watson = 1.82, ρ = −0.32). One-way ANOVA of residuals by calendar month showed no significant seasonality (*F*(11,19) = 1.11, *P* = 0.40).
