# Supplementary material for: Practice model of unit-based clinical pharmacists’ individualized daily antimicrobial use density monitoring report on antimicrobial stewardship in intensive care unit of a tertiary hospital in Guangxi, China: an interrupted time series analysis
Source: Antimicrob Resist Infect Control. 2026 Jul 2;15:96. doi: 10.1186/s13756-026-01786-9 (PMC13411574; doi:10.1186/s13756-026-01786-9)
Supplement: Supplementary file 9 — Supplementary Material 9 [file 13756_2026_1786_MOESM9_ESM.docx]

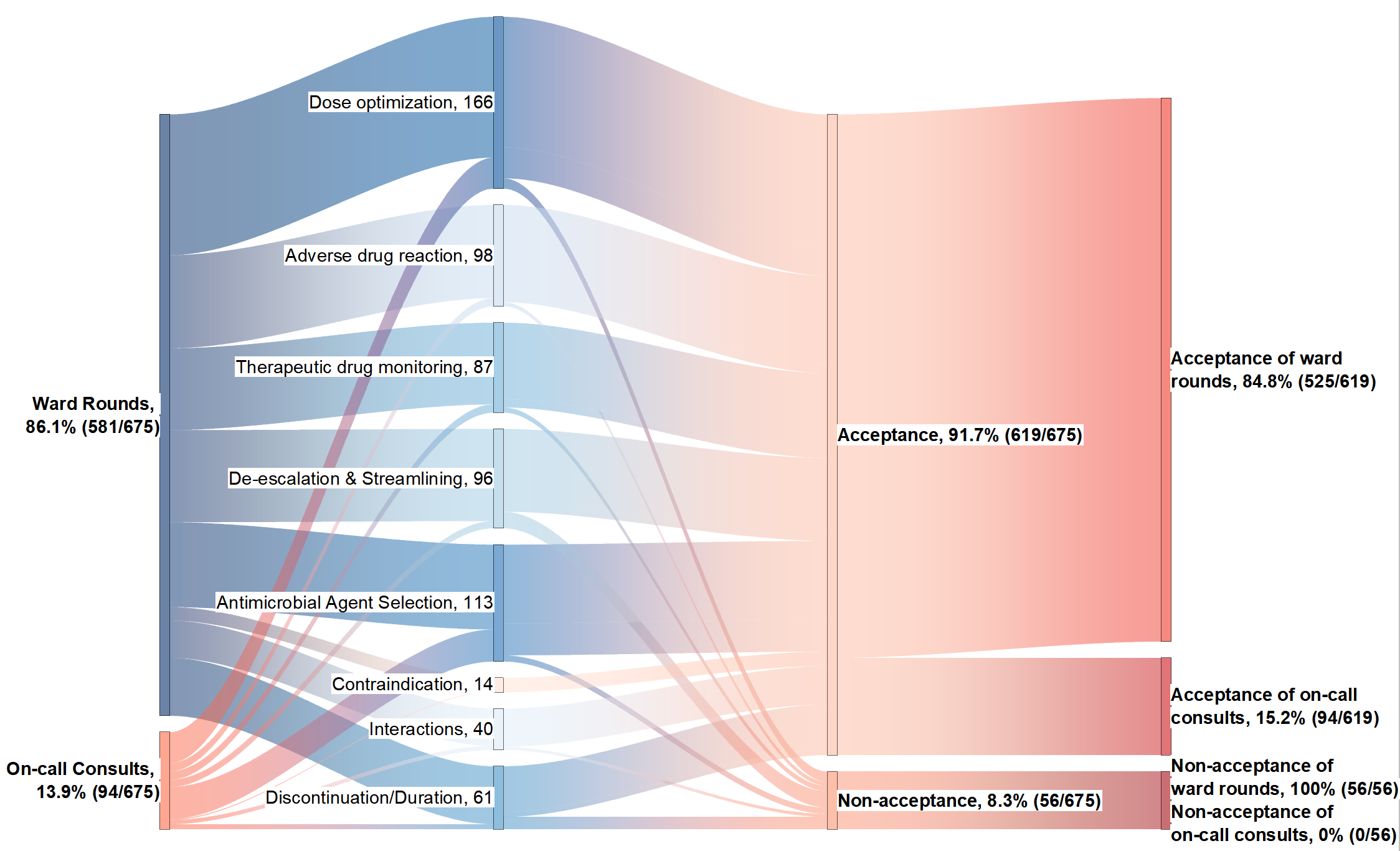


**Supplementary Figure S1.** Sankey diagram of unit-based clinical pharmacist interventions

The intervention process begins with a "Ward Rounds/On-call Consults," progresses through interventions like "dose optimization, adverse drug reaction, therapeutic drug monitoring, de-escalation/streamlining, antimicrobial agent selection, contraindication, interactions, discontinuation/duration" and concludes with outcomes such as "all acceptance/non-acceptance" and "acceptance/non-acceptance of ward rounds/on-call consults."
